# Can home care work be organized to promote musculoskeletal health for workers? Results from the GoldiCare cluster randomized controlled trial

**DOI:** 10.1186/s12913-024-12133-2

**Published:** 2025-01-07

**Authors:** Fredrik Klæboe Lohne, Marius Steiro Fimland, Javier Palarea-Albaladejo, Svend Erik Mathiassen, Andreas Holtermann, Skender Redzovic

**Affiliations:** 1 Department of Neuromedicine and Movement Science, Faculty of Medicine and Health Sciences, Norwegian University of Science and Technology, Edvard Griegs gate 8, Trondheim, 7030 Norway; 2 National Research Centre for the Working Environment, Lersø Parkallé 105, Copenhagen, DK-2100 Denmark; 3 Unicare Helsefort Rehabilitation Centre, Hysnesveien 11, Rissa, 7112 Norway; 4 Department of Computer Sciences, Applied Mathematics and Statistics, University of Girona, Girona, Spain; 5 Department of Occupational Health, Psychology and Sports Sciences, University of Gävle, Gävle, 80176 Sweden

**Keywords:** Goldilocks work, Cluster randomized controlled trial, Clinical trial, Home care, Worker health, Compositional analysis, Norway

## Abstract

**Background:**

Workers in home care have high sick leave rates, predominantly because of musculoskeletal pain. The Goldilocks Work Principle proposes that health should be promoted by a “just right” composition of work tasks. Weekly workloads differ substantially between home care workers, suggesting that certain workers may have workloads that are too high, impacting their musculoskeletal health. The aim of this study was to evaluate the effectiveness of a “GoldiCare” intervention redistributing weekly workloads to become more equal among the homecare workers. Outcomes were pain in the neck/shoulder and lower back, and the implementation of the intervention was also evaluated.

**Methods:**

A 16-week cluster randomized controlled trial was conducted with 125 workers from 11 home care units, divided into six intervention units and five control units. The operation coordinators of each intervention unit were educated in the Goldilocks Work Principle and provided with a planning tool to facilitate an even distribution of high care need clients. The control group continued their usual work. Primary outcomes were pain intensity in the neck/shoulder and lower back (0 to 10). Secondary outcomes included fatigue (0 to 10), composition of physical behaviors and postures (accelerometers), adherence to the intervention (weekly usage rates of the planning tool), and performance of the intervention (percentage of workers with an even distribution of workload).

**Results:**

The analysis showed no difference between the intervention and control groups in change in lower back pain (0.07, 95%CI[-0.29;0.43]), neck/shoulder pain (-0.06, 95%CI[-0.49;0.36]) or fatigue (0.04, 95%CI[-0.52;0.61]. No significant changes were observed in the composition of physical behaviors (*p* = 0.067) or postures (*p* = 0.080–0.131) between the two groups. The intervention was succesfully implemented in three units of the six, with adherence ranging from 82–100% across the intervention period. The remaining three units had an adherence of 0–47%. No improvement in performance was observed.

**Conclusion:**

No significant intervention effects were observed on musculoskeletal pain, fatigue, or the composition of physical behaviors and postures. The findings suggest that the intervention was not adequately implemented within the organization. Consequently, we cannot discern whether the lack of positive results were due to poor implementation or an ineffective intervention. Results thus highlight the need for a more comprehensive understanding of organizational structures within home care to facilitate more effective implementations. The hypothetical effectiveness of a fully implemented intervention remains unknown.

**Trial registration:**

Clinicaltrials.gov ID: NCT05487027, submitted: 03/08/2022.

**Supplementary Information:**

The online version contains supplementary material available at 10.1186/s12913-024-12133-2.

## Background

In 2022, Norwegian home care services provided assistance to approximately 156,000 clients aged 67 years or older [1]. With an aging population [2], home care services will play an increasingly essential role, since it is an economically sustainable alternative to institutional care [3, 4] and is often preferred by the elderly [5, 6]. To provide adequate care for the growing elderly population in Norway, more than 50% increase in the workforce will be needed by 2040, including nurses, nurse assistants and welfare nurses [7]. However, the home care sector has one of the highest rates of sick leave in Norway with a point-prevalence of 11% in 2019, almost double the national average of 6% [8]. The most prevalent cause of sick leave in Norway and globally is musculoskeletal disorders [9, 10], with pain in neck/shoulder and lower back as two of the most common reasons [11]. Extended exposure to physical work, such as standing [12], forward bending [13, 14], and arm elevation [15] is known to be associated with musculoskeletal pain, and increased risk of sick leave [16–18]. In addition, high work demands are associated with increased fatigue, which can act as an intermediary factor leading to sickness absence [19]. Many workers in home care express a desire to leave the occupation due to the high physical work demands [20]. Interventions addressing the physical work demands in home care are therefore needed, both for workers’ health and for the sustainability of the home care sector [21].

Prior research in Norwegian home care has revealed substantial differences between workers’ total weekly exposure to physical work [22]. This difference, as expressed by time spent in physical behaviors of different kinds, and with a forward bent trunk and arms elevated, suggests an uneven distribution between the workers of physically demanding tasks. Such differences in physical work likely lead to an unequal exposure to risk factors for musculoskeletal pain, exposing certain workers to a particularly high risk for neck/shoulder and lower back pain.

To improve the health of workers, the “Goldilocks Work Principle” has been suggested as an effective strategy [23]. This principle aims to redesign productive work to represent a “just right” composition of work and recovery for all workers, thereby enhancing worker health, instead of causing deterioration [24]. The Goldilocks Work Principle is a novel approach and has thus far not been tested in the home care sector. Therefore, in response to earlier research results and following consultation with home care workers and stakeholders, we developed the “GoldiCare” intervention [25]. In this intervention, “just right” was pursued by minimizing differences between workers in their weekly physical work, theoretically resulting in decreased exposure to physical work for some workers and increased for others. Thus, workers that may have too high work demands get lower demands, by redistributing some of their high demands to workers that have low work demands; and vice-versa. By redistributing work to achieve a more equal exposure among workers, the intervention seeks to improve the average musculoskeletal health across all workers, without compromising productivity.

The aim of this study is to investigate the effect of the GoldiCare intervention, in a cluster randomized controlled trial. The present study assesses the intervention effect on the primary outcome musculoskeletal health, expressed as neck/shoulder and lower back pain intensity after work. Secondary outcomes include fatigue after work, the difference in exposure to physical work between workers, and the home care units’ adherence to the intervention.

## Methods

### Trial design

This 16-week cluster-randomized controlled trial included 11 home care units allocated in an approximate 1:1 ratio between the intervention and control arms. Home care workers within a home care unit often work together on assignments, therefore, due to convenience and to avoid contamination between arms, we defined the 11 home care units as clusters. The trial was registered before enrollment (NCT05487027), and the study protocol was submitted before the first participant was enrolled [25]. The study was approved by the Regional Committee for Medical Research Ethics Central Norway (# 315556).

### Participants

All workers in a position primarily involving client care and working in a full-time equivalent (i.e.37,5 h per week) of 50% or more were invited to participate in measurements of physical behaviors and to answer questionnaires. Exclusion criteria included pregnancy, fever on the start day of measurements, allergy to the accelerometer fixation tape and physical incapabilities hindering normal home care work. The same exclusion criteria applied in the follow-up measurements. All participants read and signed a consent sheet informing of rights according to the Helsinki Declaration. A power calculation was conducted (further details in the protocol paper [25], showing that the approximately 200 participants we expected, based on previous data collections, to be able to recruit, would be sufficient to find a clinically significant effect.

While the intervention aimed to improve musculoskeletal health in the home care population, all home care workers were included, including those without pain. The intervention sought to redistribute the physically demanding work, which implies that certain workers receive less, but also certain workers receive more physically demanding work. Pain in all home care workers was therefore measured to be able to detect an increase in pain due to the redesign.

### Settings and locations

This intervention took place in Trondheim, Norway, and involved 11 of the then 13 home care units in the municipality. Two of the units did not wish to participate, due to an ongoing organizational change the two units. While home care in Norway is managed by the municipality, each unit is free to organize work according to its own preferences. This leads to 11 distinct units that, while being geographically close, sharing common objectives, and facing similar challenges, adopt different organizational structures. All home care units have one or more operations coordinator(s), who is responsible for assigning work lists. The work list is a daily schedule for client visits presented to each individual worker, defining an approximate duration of each visit and the care tasks to perform there. The assignment of work lists is influenced by several factors including: geography to ensure efficient transportation routes, the specific care needs of clients (determining whether nurses, physiotherapists, or occupational therapists are required), and political aims such as exposing clients to only few different workers.

### Activities of daily living

All home care clients in Norway are assigned an Activities of Daily Living (ADL) score which indicates the clients need for help to perform the necessary activities in daily life [26, 27]. The score is composed of several subcategories, describing all of the client’s functions. Each of the subcategories – self-care, social function, cognitive function, ability to take of own health, and domestic responsibilities – are scored by home care workers upon enrolment into home care. Each of these subcategories are scores from 1, which indicates complete independence, to 5, indicating complete dependence. The score has, till now, been used to guide economic resources and track the development of the clients, not to plan the care work. Based on research by ourselves [28] and others [29–31], we believe that the self-care subcategory of ADL can be used as an indicator of the demands in home care work, and we used it for that purpose in the present study.

### GoldiCare intervention

Prior to the intervention, Tjøsvoll et al. [22] found large differences between workers at six home care units in Trondheim in weekly physical work. This notion was corroborated by Liaset et al. [32], in workshops with home care workers where the potential for interventions was explored. One important area suggested for improvements was the distribution of demanding work lists, as workers expressed that easy and strenuous work lists were unequally distributed between workers. Based on this observation, we designed an intervention aiming at achieving a more even weekly distribution between workers in how often they visited clients with high care demands. An even weekly distribution, as opposed to a daily, was chosen for feasibility reasons, as it gave operations coordinators more flexibility. Balancing the work lists between workers would theoretically lead to reduced differences in physical work demands between workers and approach a “just right” distribution of demanding work for each worker.

In this intervention we defined a client needing high care as having an ADL self-care score of ≥ 4 (hereby referred to as “high ADL clients”) [27, 28]. Based on the number of high ADL clients on the work lists, each workday was categorized as “high” (highest number of high ADL clients), “medium” (medium number of high ADL clients), or “low” (lowest number of high ADL clients) with definitions of “high”, “medium” and “low” based on the operations coordinator’s experience and the unit’s total number of high ADL clients, which differed between the units. An even weekly distribution of work lists (i.e. an equal number of “high” and “low” workdays between workers was facilitated by supplying the operations coordinator with a “GoldiCare tool”. A similar tool was first developed in a Goldilocks Work intervention for industry [33] and later adapted to home care in a feasibility study before being further adapted to the current intervention [25]. The “GoldiCare tool” gave the operations coordinator an overview of the distribution of “high”, “medium”, and “low” work lists among workers during the process of assigning lists to individual workers and helped the coordinator to achieve an even weekly distribution across workers between shifts of “low” and “high”, with “medium” being considered neutral as they had a balanced number of demanding clients.

The tool was introduced to the home care units during two meetings with the operations coordinators. At the first meeting, all operations coordinators employed at the intervention units participated, and they received an educational session about the Goldilocks Work Principle, the current intervention, and an introduction to the GoldiCare tool. The second meeting was arranged to give a thorough and personalized introduction to the GoldiCare tool for each individual unit’s operations coordinator. During this meeting, the clients to be included in the “high” work lists, i.e. those with a high ADL self-care score, were identified and the GoldiCare tool was customized to the specific requirements of the unit.

### Measurements

#### Baseline characteristics

Before entering baseline measurements, all participants were asked to complete a questionnaire, designed specifically for intervention (Additional file 1), addressing their age, gender, country of birth, how long they had been working in home care, their current position in the home care unit, and working time in percent of full time. Further, they were asked about selected items from the Norwegian version of the Copenhagen Psychosocial Questionnaire III (COPSOQ III) [34, 35]. In the present paper we used item JU4, “Is the work distributed fairly?”, with answer options “to a very large extent”, “to a large extent”, “somewhat”, “to a small extent”, “to a very small extent”. They were also asked about several health indicators including an ad hoc developed self-assessed health question, rated on a 4-point scale from “bad” to “very good”, their current workability relative to their all-time best, with a 0 indicating “unable to work” and 10 indicating “all-time best ability”, and their sickness absence history by the question “have you been on sickness absence the previous 12 months?”. If yes, they were asked “What is your total sick leave in the last 12 months?”, with alternatives more or less than 2 weeks.

#### Primary outcomes

Musculoskeletal pain was assessed by having workers rate their pain on a 0 to 10 scale, with a 0 indicating “no pain”, and 10 indicating “severe” pain. At the end of each working day, both during baseline (August 2022 – November 2022) and follow-up (March 2023 – May 2023) measures, the worker responded to two questions i.e. “How much pain did you have in the lower back at the end of this working day?” and “How much pain did you have in the neck/shoulder at the end of this working day?”. The questions were part of an activity diary, further described below (Additional file 2).

### Secondary outcomes

#### Fatigue

Intervention effects on fatigue was assessed by a question developed ad hoc “How tired were you at the end of this working day?”. Similar to the musculoskeletal pain questions, fatigue was rated on a 0 to 10 scale, with larger numbers corresponding to more fatigue, and measured at the end of every working day during both baseline and follow-up.

#### Physical behavior and postures

Physical behaviors were measured using three Axivity AX3 accelerometers (Axivity Ltd, Newcastle upon Tyne, UK) attached to the thigh, upper back and upper arm [36, 37]. Data were collected for up to seven consecutive days, both during baseline and follow-up. The participants also filled in an activity diary noting the time of, 1) waking up, 2) starting to work, 3) finishing work, 4) going to bed. After seven days, participants returned the accelerometers and the activity diary in a closed envelope in a box placed at the workplaces. Accelerometer data were processed into time spent at work in different physical behaviors and with arm elevation and forward bending (collectively referred to as “postures”) using the MATLAB software Acti4 [36, 38]. Worktime was identified using the diary. Data describing time spent in behaviors during worktime are compositional [39, 40]. The parts making up the total worktime are co-dependent and convey relative information. The use of time at work was described using three different compositions. Physical behavior was described by the composition of time spent sedentary (lying down and sitting), standing (standing still and standing with small movements), and being active (walking, running, stair climbing and cycling). Postures were categorized as the composition of sedentary time (defined as above), upright time with arms elevated and upper body forward bent equal to or under a threshold value (60° for arm elevation, 30° for forward bending) and upright time with arms elevated or upper body forward bent above the threshold value. These compositions have been used previously to investigate associations with neck/shoulder pain and lower back pain [41].

#### Adherence and performance

The units’ adherence to the intervention and how well it performed i.e. how well the operations coordinators managed to distribute work lists evenly between workers, were registered throughout the intervention period in the GoldiCare tool. To quantify adherence, we noted the number of weeks during the intervention during which operations coordinators used the GoldiCare tool, relative to the total number of possible weeks. For a week to be counted, at least three workdays had to be registered in the tool. To assess performance, we used the percentage of workers who had a “just right” distribution of physically demanding shifts (i.e., an even distribution of “low”, and “high” worklists, after the intervention, compared to baseline).

#### Randomization and blinding

No blinding took place in this intervention, as all intervention units participated in the delivery of the intervention. Randomization of units to the intervention and control groups was conducted by the unit for Applied Clinical Research at the Norwegian University of Science and Technology (https://www.klinforsk.no/). To ensure similar number of participants in each group, the randomization was stratified by the number of participants at each unit. Allocation of the units took place after the baseline measurements.

### Statistical analysis

Descriptive characteristics of participants' baseline status were reported by the number of participants or occurrences, in absolute numbers and as a percentage of the total. Continuous variables were summarized by the mean and standard deviation (SD). Average time spent in physical behaviors and postures were described by geometric means normalized to an average home care working day (450 min, 7.5 h) and expressed as percentage of time.

Intervention effects on questionnaire items (i.e. musculoskeletal pain and fatigue) were assessed using linear mixed models, with the daily score of pain or fatigue (from 0 to 10) as the dependent variable(s). Fixed effects were time (baseline or follow-up) and group (control or intervention) and the interaction between time and group. Participant ID nested within unit ID was included as random effects to account for the clustered structure defined by the home care units and the repeated measurements for each participant. Results of the analyses are reported as estimated means for each group at each time point, along with the estimated effect of the intervention and the corresponding 95% confidence intervals.

Linear mixed models were also used to investigate the effects of the intervention on weekly differences in workload between workers, using three response variables, 1) the physical behavior composition (sedentary, standing, active); and 2) the two posture compositions (sedentary, upright ≤ threshold, upright > threshold) related to 60° arm elevation and 30° forward trunk bending. Following the recommended compositional data approach, compositions are represented through orthonormal log-ratio (olr) coordinates (a.k.a. isometric log-ratio, ilr, coordinates) [39, 40, 42]. Each of the 3-part compositions described above resulted in two olr coordinates used as response variables in mixed models. For the purpose of this analysis, it is irrelevant what particular olr representation is chosen. To manage the fitting of the multivariate response model, we followed the usual procedure to turn it into an ordinary single response model fit. That is, the two olr coordinates for each individual were stacked into a single response variable and an indicator variable distinguishing them was added as covariate in the model. In addition, all these models included time (baseline or follow-up), group (control or intervention) and the interaction between them (time x group) as fixed effects. Random effects included home care unit ID with participant ID nested within. In order to statistically test whether changes in differences between workers from baseline to follow-up differed between the intervention and control groups, two versions of the above models were used: one using a homogeneous, i.e. equal variance structure between groups (null hypothesis); and another using a heterogeneous variance structure, i.e. allowing different variance by group and time (alternative hypothesis). The significance of the difference between variance structures was then statistically tested using the likelihood ratio test. A significant result would thus suggest meaningful differences in variability between control and intervention groups in the physical behavior and posture compositions.

To visualize the data and to facilitate understanding of the variability and associations for weekly averages of physical behaviors and postures, we produced compositional biplots based on principal component analysis (PCA) [43]. PCA is a widely used technique to project multivariate data onto fewer dimensions, which facilitates understanding of trends, patterns and relationships across variables and observations [44]. In brief, PCA generates new variables (the principal components, as many as original variables) that are linear combinations of the original ones, successively accounting for decreasing amounts of the total variance of the data. Thus, the first two principal components (PC1 and PC2) account for the largest fraction of such variability and are commonly used to produce a two-dimensional diagram called a biplot, which simultaneously represents individuals and variables. In the present case, the previously described compositions are transformed using a centered log-ratio (clr) transformation before processed using ordinary PCA and biplots [43, 45]. In the current study, given that the compositions consisted of only three parts, PC1 and PC2 are in fact enough to account for 100% of the total variability.

For interpretation, note that in the compositional biplot, individual workers are represented by points (i.e. points close to each other shows that those individuals have similar compositions). The parts of the composition are represented by arrows from the origin. The length of the arrow informs about the relative contribution of a compositional part to the total variability. The direction of the arrowheads indicates increased time of the respective parts of the composition and, hence, points located near the arrows in the biplot will correspond to individual compositions led by those parts. The distance between two arrowheads indicates the association (in terms of proportionality) between the corresponding parts, so the closer the arrows, the stronger the association between them. In contrast, arrowheads far from each other suggest that parts are more independent. Unlike ternary plots of the original observations (where visual distances can be difficult to translate into actual similarities between individuals), compositional biplots allows representation in an ordinary scatterplot-like graph more than three parts and facilitate visualization and interpretation of the variability of the study population and any subgroups therein, and the relationships with for all variables. The interested reader is referred to the specialized literature for more details [44, 46, 47].

All analyses were conducted in R (version 4.3.1) for statistical computing (R Foundation for Statistical Computing, Vienna, Austria [48]), using the packages “lme4” [49] and “nlme” [50] for mixed modelling and significance testing, the package “coda.base” [51] for log-ratio coordinate representations, and “FactoMineR” [52] and “Factoextra” [53] for PCA and biplot graphical display. Statistical significance was concluded for test *p*-values below the 0.05 level.

### Sensitivity analysis

To investigate whether units with high adherence to the intervention had different effects of the intervention than all units together, we conducted a per-protocol analysis by stratifying the units into those with less than 80% adherence and those with more than 80%, based on percentage of weeks they used the GoldiCare tool. We then conducted analyses using the same models as described above on the units with more than 80% adherence to the intervention.

## Results

**Fig. 1:**
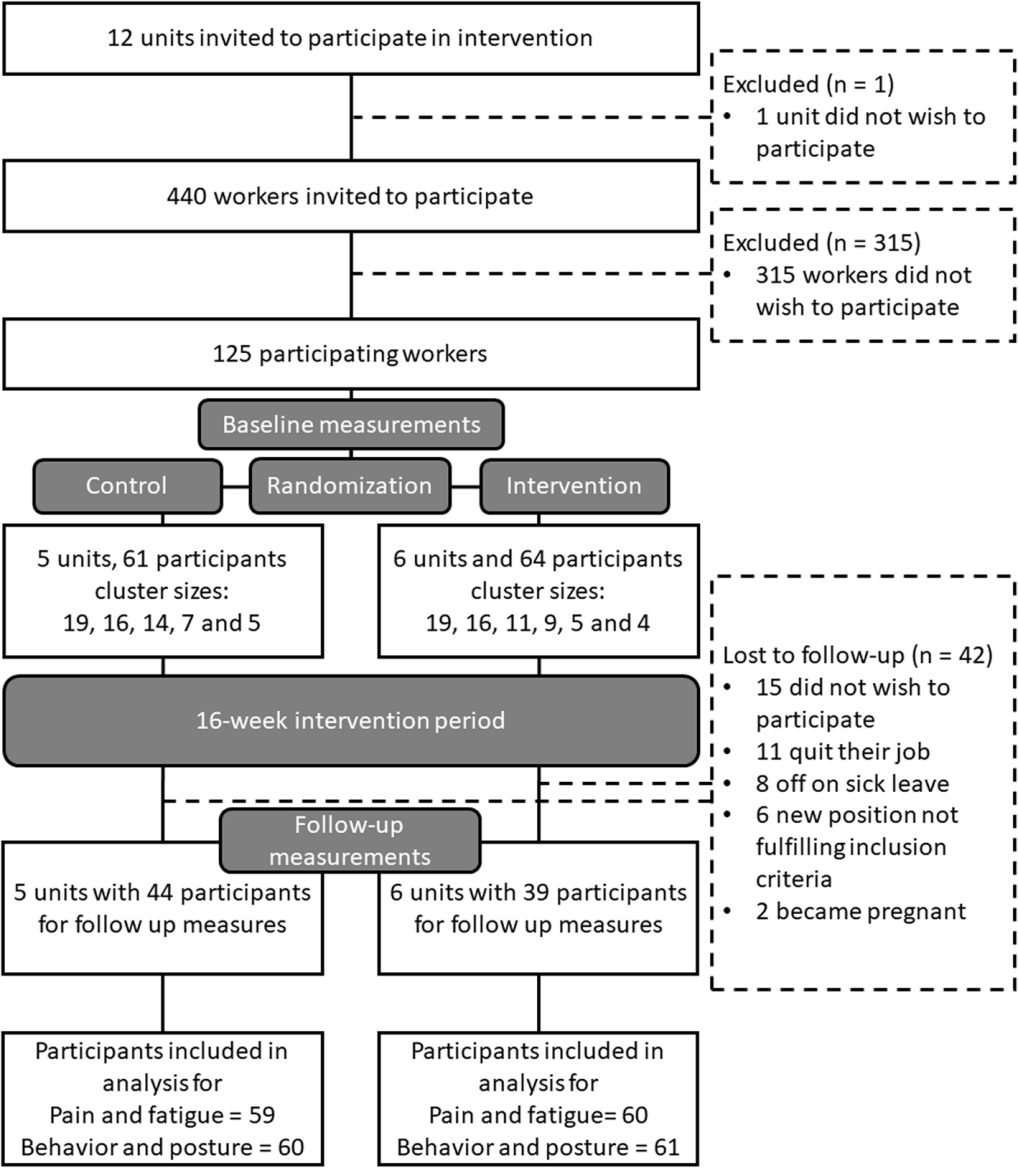
Flowchart of participants in accordance with recommendation by the “CONSORT 2010 statement: extension to cluster randomized trials” [54]

The participating eleven units had approximately 440 workers fulfilling the inclusion criteria of working 50% full-time or more. Of these, approximately 30% (131 workers) agreed to participate. Six participants did not complete the baseline measures. The randomization allocated five units with 61 participants to the control arm and six units with 64 participants to the intervention arm. At the follow-up measurements, 42 participants were lost. The most common reasons being not wanting to participate further, having ended employment, and off on sick leave. Due to certain participants not completing either accelerometer or pain/fatigue data, the resulting number of participants in an analysis differed between outcomes. Figure 1 illustrates the flow of units and participants.

### Baseline characteristics

The intervention and control groups were similar in being predominantly female, and mainly consisting of nurses and nurse assistants. Almost all participants worked 100% full time, and more than 80% had experienced sick leave in the previous 12 months. Fifty percent of the workers in the intervention group felt that work was distributed fairly and 35% in the control group. In the intervention group, approximately 75% reported a self-rated health of “good” or “very good”. The corresponding percentage in the control group was 65% (Table 1).

**Table 1:** Descriptive data for the population included in the randomized controlled trial, separated into intervention and control group

	Intervention (*n* = 64)	Control (*n* = 61)
Age (years), mean (SD)	31.6 (10.1)	32.3 (10.5)
Gender (female), n (%)	42 (81.3%)	45 (73.8%)
BMI (kg/m^2^), mean (SD)	26.9 (5.4)	27.4 (5.3)
Years worked in home care, mean (SD)	4.7 (5.1)	5.3 (6.3)
Work Ability Index (0-10), mean (SD)	8.4 (1.5)	8.6 (1.4)
Job title		
Nurse assistant, n (%)	21 (33.3%)	23 (37.7%)
Nurse, n (%)	24 (38.1%)	20 (32.8%)
Occupational therapist, n (%)	5 (7.9%)	8 (13.1%)
Social worker, n (%)	7 (11.1%)	3 (4.9%)
Other, n (%)	6 (9.5%)	7 (11.5%)
Percentage full time equivalent		
100%, n (%)	51 (81.0%)	52 (85.2%)
80%-99%, n (%)	7 (14.3%)	5 (8.2%)
<80%, n (%)	3 (4.7%)	4 (6.6%)
Country of birth		
Scandinavia, n (%)	57 (89.1%)	53 (88.3%)
Outside Scandinavia, n (%)	7 (10.9%)	7 (11.7%)
Sick leave last 12 months (yes), n (%)	52 (81.3%)	50 (83.3%)
Of which more than 2 weeks, n (%)	21 (40.3%)	24 (48.0%)
Distribution of work		
Fair (0 – 1), n (%)	30 (50.0%)	21 (35.0%)
Quite fair (2), n (%)	25 (41.7%)	27 (45.0%)
Unfair (3-4), n (%)	5 (8.3%)	12 (20.0%)
Self-rated health		
Very good, n (%)	6 (9.5%)	11 (18.3%)
Good, n (%)	42 (66.7%)	27 (45.0%)
Bad, n (%)	15 (23.8%)	22 (36.7%)

### Outcomes

#### Musculoskeletal health and fatigue

We found no relevant differences in pain and fatigue between the intervention and the control groups at baseline, and no significant intervention effect on neck/shoulder pain (−0.06, 95% CI [−0.49 to 0.36]), lower back pain (0.07, 95% CI [−0.29 to 0.43] or fatigue (0.04, 95% CI [−0.52 to 0.61]) (Table 2).

**Table 2:** Intervention effects on musculoskeletal pain in neck/shoulder and lower back, as well as on fatigue (0-10), presented as mean values at baseline and follow-up for the intervention and control group, as well as the intervention effect

		Intervention group		Control group		Intervention effect	
Outcome	Timepoint	Mean	95% CI	Mean	95% CI	Mean	95% CI
Neck/shoulder pain	Baseline	1.60	1.01 to 2.19	1.64	1.01 to 2.26	-0.06	-0.49 to 0.36
	Follow-up	1.62	1.01 to 2.23	1.72	1.09 to 2.35		
Lower back pain	Baseline	1.27	0.62 to 1.92	1.60	0.90 to 2.29	0.07	-0.29 to 0.43
	Follow-up	1.39	0.72 to 2.05	1.64	0.94 to 2.34		
Fatigue	Baseline	3.44	2.76 to 4.12	3.54	2.83 to 4.26	0.04	-0.52 to 0.61
	Follow-up	3.65	2.95 to 4.35	3.71	2.98 to 4.43		

#### Physical behaviors and postures

Table 3 presents the average time spent in physical behaviors and postures for both the control and intervention group at baseline and follow-up, normalized to a standard 450-min home care workday and as percentage. The averages show that most of the workday was spent in sedentary behaviors. Time spent upright, including both standing and active periods, totals about three hours per day. For the majority of this time participants were standing, with less than one hour spent in active movements. Furthermore, of the time spent upright, workers were bent forward more than 30° for approximately half an hour on average and had an arm elevation above 60° for between 6.9 to 8.6 min.

**Table 3:** Geometric mean of time spent in physical behaviors and postures, normalized to an average workday (450 min). Values are presented as averages in minutes and percentage of the workday for the control and intervention groups at baseline and follow-up

		Physical behaviors (minutes (%))			Postures (minutes (%))	
Group	Time point	Sedentary	Standing	Active	Forward bending > 30°	Arm elevation > 60°
Control	Baseline	260.3 (57.9)	145.6 (32.4)	44.0 (9.8)	28.7 (7.1)	6.4 (1.4)
	Follow-up	272.3 (60.5)	135.9 (30.2)	41.8 (9.3)	32.1 (6.8)	6.7 (1.5)
Intervention	Baseline	236.7 (52.6)	161.3 (35.8)	52.0 (11.6)	36.2 (8.1)	7.9 (1.8)
	Follow-up	260.2 (57.8)	140.9 (31.3)	49.0 (10.9)	28.7.5 (6.4)	8.1 (1.7)

**Fig. 2:**
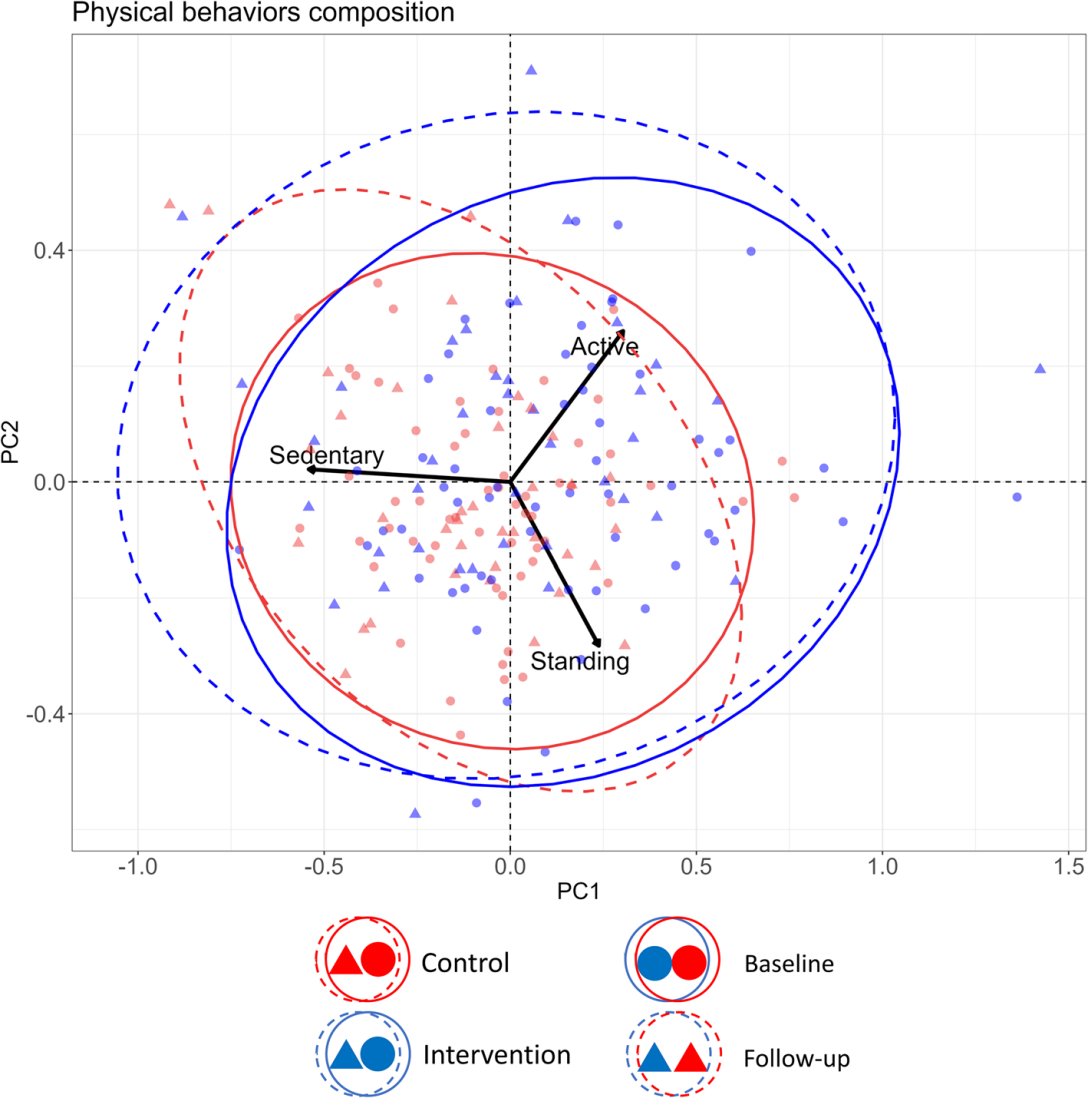
Compositional biplots illustrating the composition of physical behaviors of the intervention (blue) and control group (red) at baseline (fully drawn curves) and after the intervention (dashed curves). The center of the graph (x = 0, y = 0) represents the average composition of the groups. Individual points represent each worker’s weekly mean composition (sedentary, standing, and active). Ellipses illustrate 95% confidence regions for the variance within each group and timepoint (the tighter the ellipse the lower the variability). Arrowheads indicate the most prevalent posture in the respective direction

We found marginally no statistically significant intervention effect on the structure of variability between groups of workers (likelihood ratio = 7.14, *p* = 0.067). The compositional biplot shown in Fig. 2 provides additional insight into patterns of the variability in physical behaviors for the intervention and control groups at baseline and follow up. The 95% confidence ellipses were added to facilitate interpretation and assessment of within-group variabilities. The figure illustrates that, in general, more variability was apparent in both groups at follow-up compared to baseline, and that the intervention group had a somewhat higher variability than the control group both at baseline and follow-up. It is evident from the biplot that more workers with an outlying composition of physical behaviors were present in the intervention group than in the control group, contributing to increase the intervention group variability particularly in the direction of less sedentary and more active time (right-hand side of the graph).

**Fig. 3:**
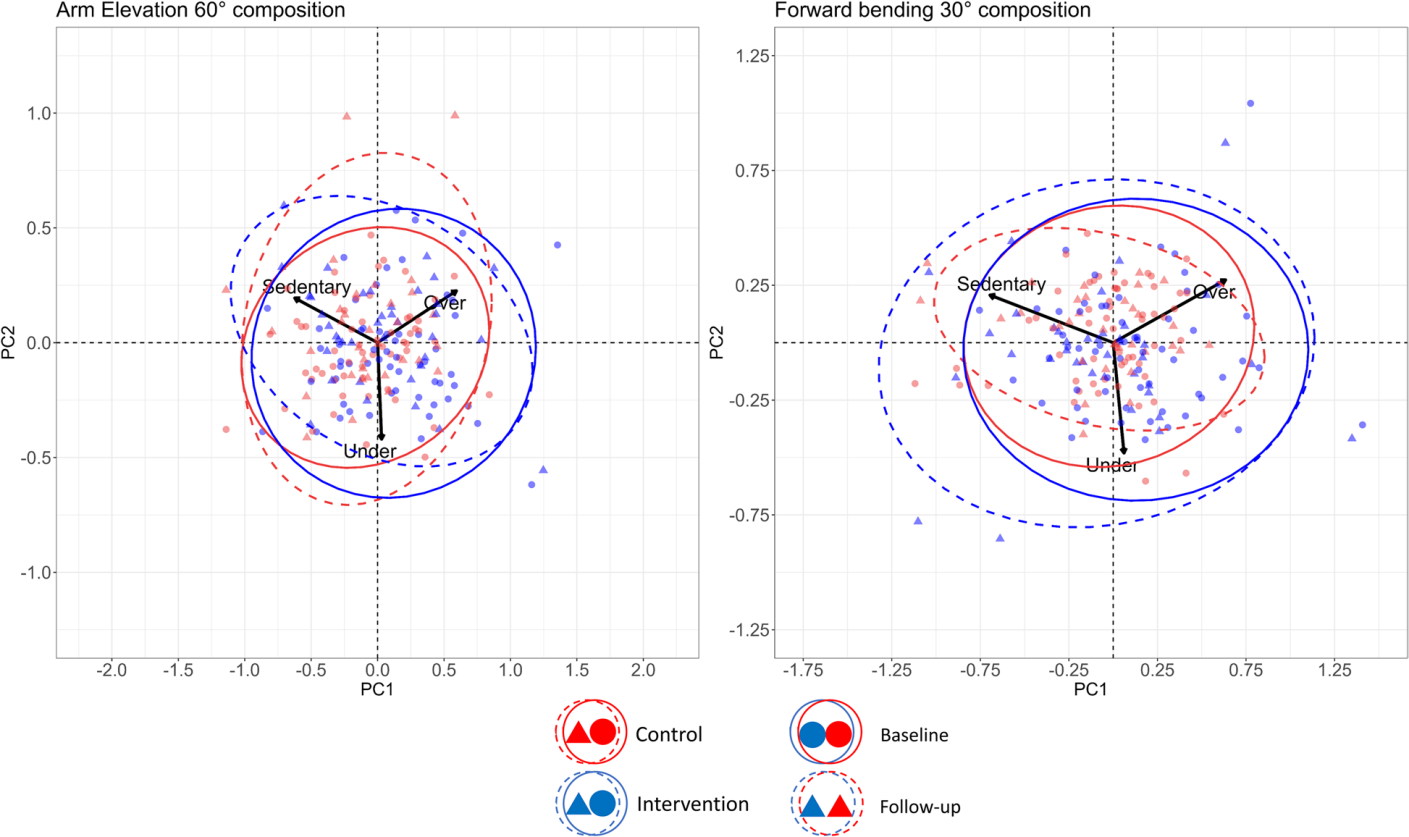
Compositional biplots illustrating the composition of arm elevation (left panel) and trunk forward bending (right panel) of the intervention (blue) and control group (red) at baseline (fully drawn curves) and after the intervention (dashed curves). The graph centers (x = 0, y = 0) represent the average composition of the groups. Individual points represent each worker’s weekly mean composition of arm elevation (sedentary, upright ≤ 60°, upright > 60°) and trunk forward bending (sedentary, upright ≤ 30°, upright > 30°). Ellipses represent 95% confidence regions, illustrating the variability within each group and timepoint (the tighter the ellipse the lower the variability). Arrowheads indicate the most prevalent posture in the respective direction

The variability between workers in time spent with arm elevation and trunk forward bending was marginally not statistically different between the intervention and control group (likelihood ratio = 5.62, *p* = 0.132 and likelihood ratio = 6.74, *p* = 0.080, respectively). The biplot in Fig. 3 presents a visualization of the observed weekly work patterns. The variability was similar for the two groups at both time points for the arm elevation composition, but the intervention group trended towards compositions with less sedentary time (lower right corner), compared to the control group. The arrow lengths were approximately equal for all postures, indicating that all postures contributed similarly to the total variability. The forward bending composition suggested more variability observed in the intervention group. Furthermore, the biplot clearly indicated the intervention group to include more outlying participants, especially in the direction of less sedentary time and with trunk bent forward more than the threshold of 30°. Arrow lengths indicate that most variability is observed for sedentary behavior and above the 30° threshold.

#### Adherence and performance

**Fig. 4:**
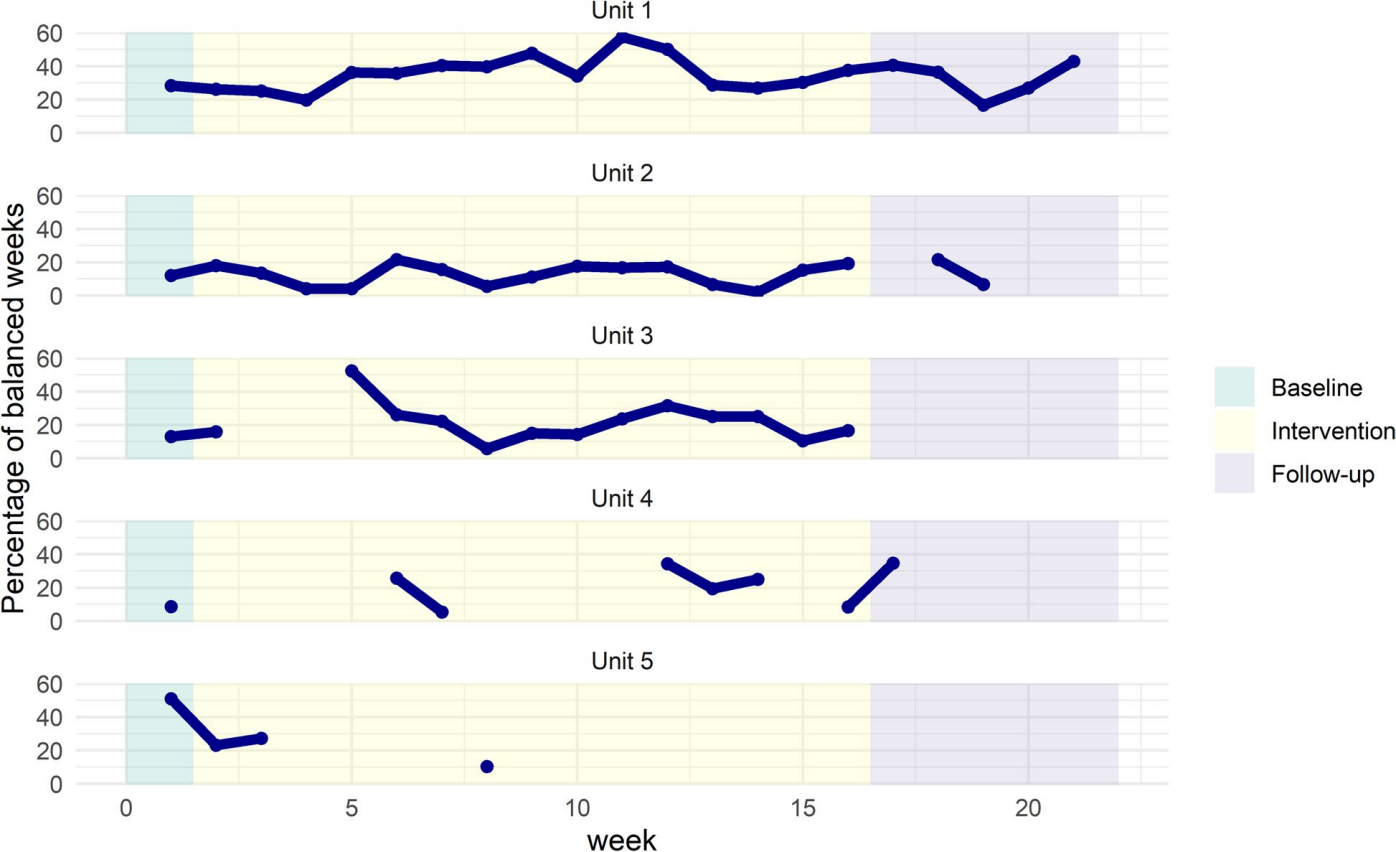
Adherence and performance of the intervention. Percentage of workers who received a balanced week (i.e., even distribution of light, heavy and medium shifts throughout the week). Breaks in lines are due to not using the tool for two weeks consecutively. Different endpoints of lines are due to different follow-up measure timepoints, as the units used the tool until the participants belonging to the respective units finished measurements. One unit excluded due to dropping out

We found large differences between the units in adherence to the intervention (Fig. 4). Three out of the six units had high adherence to the intervention, using the GoldiCare tool 82%, 95%, and 100% of the weeks. Amongst the remaining three, one unit dropped out of the intervention, and two had 22% and 47% adherence rates. Performance, i.e. the percentage of workers who got a balanced work week during the intervention period according to the GoldiCare tool, varied between weeks and did not show any improvement over time, even in units that consistently used the tool.

### Sensitivity analysis

The sensitivity analysis including 28 participants from the high adherence units, i.e. units that used the GoldiCare tool for 80% or more of the intervention weeks, still showed a non-significant intervention effect. The effect on musculoskeletal pain and fatigue suggested that differences compared to the control units were trivial even for the high adherence units compared to the control group units (Additional File 3, Table S1). We observed no significant results towards a more even composition of physical behaviors (likelihood ratio = 2.14, *p* = 0.543) (Additional File 3, Figure S1). For arm elevation there was no significant change towards a more even distribution (likelihood ratio = 5.93, *p* = 0.115) for the high adherence units compared to the control group (Additional File 3, Figure S2). In contrast, forward bending showed a significant increase of the variability in the high adherence intervention group (likelihood ratio = 10.96, *p* = 0.012), indicating a negative effect of the intervention (Additional File 3, Figure S2). It should be noted that statistically significant differences were largely driven by outlying observations.

## Discussion

This is the first study to evaluate a Goldilocks Work intervention in home care. The aim of the intervention was to improve musculoskeletal health among the workers, by reducing the difference between workers’ exposure to physical work through a more even distribution of high care need clients among workers. We did not find any intervention effect on the outcomes i.e., musculoskeletal pain, fatigue, physical behaviors, and postures. The adherence to the intervention was poor in three of the six intervention units.

Only a few interventions in home care have been reported [21], let alone interventions that can be reasonably compared with the GoldiCare intervention. Both Czuba et al. [30] and Leff et al. [55] reorganized how schedulers, i.e., operations coordinators in the present study, distributed the most demanding clients. Unfortunately, none of them used a randomized controlled trial design; both used a pre-to-post design. Furthermore, they both combined the reorganization with other intervention elements, making it difficult to isolate the effect of the reorganization per se. Czuba et al. [30] conducted an intervention limiting how often home care workers visited the most demanding clients. They found a significant lower fatigue rating on workdays containing at least 50% high demand clients, compared to workdays with less than 50%. Furthermore, they found an increased likelihood of reporting pain at the end of the workday with larger numbers of demanding clients. Leff et al. [55] also developed an intervention addressing how schedulers distributed the most physically demanding clients. They found a decrease in the number of workers with shoulder and back injuries; however, the study design makes it impossible to isolate the effect of redistribution of demanding clients from other elements of the interventions. Further, the effect was not statistically tested. A systematic review from 2022 by Gebhard et al. [21] investigated interventions in the home care sector in order to suggest recommendations for future interventions. Out of 19 publications, six had pain as an outcome. However, none of them intervened on the organization of physical work demands. Furthermore, four of the six publications reported no intervention effect on pain [56–59]. Two studies succeeded in improving musculoskeletal health, where one intervened on the leisure time of workers [60], and one implemented lumbar support for workers [61]; thus none of these are comparable with the current intervention.

As the intervention did not show any obvious effects on the primary- and secondary outcomes, an evaluation of the program logic and implementation plan is appropriate [25]. Facilitators and barriers to the implementation are appropriately assessed by using semi-structured interviews [62], and will therefore be assessed in a separate process evaluation paper (Fischer H, Lohne FK, Fimland MS, Redzovic S: "It's a good idea, but…" A Qualitative Evaluation of the GoldiCare Intervention in Norwegian Home Care Services, forthcoming). Thus, the present discussion will deal with the program logic [25]. In short, the program logic was based on the observation that home care workers differed considerably in weekly physical work exposure, along with a high prevalence of musculoskeletal pain in the neck and shoulder [22]. We therefore proposed that musculoskeletal health could improve among the workers by reducing the highest work load, and increasing the lowest, i.e. redistributing clients among workers to be more “just right” in terms of a fair and even distribution of workload between workers [25]. The intervention was to be implemented using the GoldiCare tool, used by the operations coordinator to distribute the physically demanding worklists more evenly. Thus, the success of the intervention was dependent on the operations coordinators and how well they followed the intervention protocol, including the extent to which they succeeded in distributing worklists more evenly.

The adherence to the intervention differed vastly between the intervention units, as three units utilized the tool for more than 80% of the weeks, and the remaining three used it for 47%, 22% and 0%. All participating units received identical training and introduction to the intervention. The observed differences in adherence therefore suggest that organizational variations among units may have had an impact on the implementation of the intervention. Factors having a likely influence on intervention success may include the number of operations coordinators, and the total workload and/or number of workers per operations coordinator. Furthermore, the performance, as gauged by the proportion of workers with a “just right” work week, varied week-to-week for all participating units and did not exhibit any trend towards improvement (Fig. 4). This might point to organizational elements out of the operations coordinators’ control, such as workers’ preferences for specific worklists, workers swapping worklists, and variations in qualifications among workers. Understanding how organizational structures might have acted as facilitators and barriers to the intervention adherence and performance will be explored in the process evaluation. Considering the low adherence of the units to the intervention and that no improvement in performance over time was found, it agreed with the program logic that we did not find any obvious differences between the groups in how physical work demands changed, and thus no difference in musculoskeletal health. It should, however, be noted that due to the low adherence, we cannot discern whether the intervention would have been successful in improving musculoskeletal health of the home care workers had it been successfully implemented.

The sensitivity analysis on units with the highest adherence, showed a statistically significant change in the intervention group of variability between workers for forward bending compositions. However, results remain uncertain due to few participating workers. Further, given that no unit demonstrated an improved balance of work lists, uncertainty remains about whether the statistically significant result for the forward bending composition is attributable to the intervention itself or to external factors, especially since the results suggested a negative intervention effect i.e., larger variance between workers.

While neck/shoulder pain and lower back pain were extensively prevalent, being reported on approximately 50% of the investigated working days, the reported intensity of pain was relatively low with most pain ratings showing mild pain. This low baseline pain intensity inherently limits the potential for notable improvements at follow-up, including achieving a clinically significant effect on pain, i.e. a two-point decrease on the 0-10 scale [63]. A successful intervention may have lowered the average musculoskeletal pain, but likely only to a limited extent, and the home care workers would still be among the groups with the highest musculoskeletal pain in Norway. To more pervasively influence the musculoskeletal pain in this group, more radical interventions and organizational changes are likely required. Further, it is possible that a similar intervention could have led to a larger effect in a working population experiencing higher intensities of musculoskeletal pain, if successfully implemented.

### Strengths and weakness

A major strength in this study is the comprehensive approach of mapping work demands and employing a participatory approach, culminating in a feasibility study (unpublished). This participatory approach ensured that the intervention was not only based on scientific research, but also considered insights and preferences of home care workers. Furthermore, using technical measurements to assess physical behavior and demanding postures allowed for a valid evaluation of the distribution of weekly physical workload. Additionally, assessing musculoskeletal pain at the end of every workday during the measurements period reduced the risk of recall bias, and increased statistical power in the analysis. Also, the utilization of ADL in the intervention as a proxy for the exposure to physical work associated with handling clients is a strength. ADL is based on a WHO framework [26], which makes the intervention and its’ results nationally and internationally applicable.

A major weakness of this study was the low participation rate, amplified by a high dropout, which led to reduced statistical power and increased risk of selection bias. Especially in the follow-up measurements, the dropout may have resulted in an overrepresentation of workers experiencing high pain intensities and schedules with high workloads, as they would likely have the highest interest in further participation. An additional selection bias may be due to the healthy worker effect. Only workers with low pain intensities stay active at work, while the workers suffering the most are either on sick leave or have quit. Further, since the number of high ADL clients required to classify a work list as representing “high” demands was determined by each unit’s operations manager, the definition of a ‘”high” list was not uniform across the participating units. This inconsistency challenges comparisons between units and complicates the potential for applying the same intervention in other home care units. Lastly, the low average pain scores at baseline, with most workers reporting many days of zero pain, also limited the extent of possible improvement in musculoskeletal health.

## Conclusion

The GoldiCare intervention aimed to promote musculoskeletal health among home care workers by reducing differences between workers’ exposure to weekly physical work. However, the intervention did not have an effect on musculoskeletal health. The intervention showed to be inadequately implemented in the organization, as only half of the participating units adhered to the intervention. Further, operations coordinators did not manage to distribute the weekly worklists more evenly between workers. Due to this unsuccessful implementation, results expectedly suggested a lack of changes in exposure to weekly physical work among the home care workers, and thus a change in their musculoskeletal health status could not be expected. Given the inadequate implementation, we cannot draw conclusions as to whether the intervention would be effective in having positive effects on musculoskeletal health in a more favorable context. Therefore, future research should focus on improving the implementation of interventions at the organizational level focusing on improved musculoskeletal health in home care.

## Supplementary Information

Additional file 1. GoldiCare – Questionnaire. Translated (Norwegian to English) baseline characteristics questionnaire.

Additional file 2. Activity diary. Translated (Norwegian to English) activity diary which was used by participants to note start of periods (work, leisure, sleep, waking up) and their pain in neck/shoulder, lower back and fatigue at the end of the workday.

Additional file 3. Sensitivity analysis results. Results from sensitivity analysis of pain and fatigue, as well as visualization of behaviors and posture compositions from sensitivity analysis.

## Data Availability

The datasets used and analysed during the current study are available from the corresponding author upon reasonable request.

## Abbreviations

PCA: Principal component analysis
ADL: Activities of daily living
Clr: Centered log-ratio
Olr: Orthonormal log-ratio
COPSOQ III: Copenhagen Psychosocial questionnaire III

## References

[1] Helsedirektoratet. Kommunale helse- og omsorgstjenester 2022: Helsedirektoratet; 2023. Available from: https://www.helsedirektoratet.no/rapporter/kommunale-helse-og-omsorgstjenester-2022.

[2] United Nations, Department of Economic and Social Affairs, Population Division. World Population Prospects 2022: Summary of Results. 2022;UN DESA/POP/2022/TR/NO. 3.

[3] Marek KD, Stetzer F, Adams SJ, Popejoy LL, Rantz M. Aging in place versus nursing home care: comparison of costs to Medicare and Medicaid. Res Gerontol Nurs. 2012;5(2):123–9. 21846081 10.3928/19404921-20110802-01

[4] Chappell NL, Dlitt BH, Hollander MJ, Miller JA, McWilliam C. Comparative costs of home care and residential care. Gerontologist. 2004;44(3):389–400. 15197293 10.1093/geront/44.3.389

[5] Wiles JL, Leibing A, Guberman N, Reeve J, Allen RES. The meaning of “aging in place” to older people. Gerontologist. 2011;52(3):357–66. 21983126 10.1093/geront/gnr098

[6] Moe A, Hellzen O, Enmarker I. The meaning of receiving help from home nursing care. Nurs Ethics. 2013;20(7):737–47. 23625732 10.1177/0969733013478959

[7] Zhiyang J, Kornstad T, Stølen NM, Hjemås G. Arbeidsmarkedet for helsepersonell fram mot 2040. 2023.

[8] NAV. Sickness absence statistics (translated). In: Administration NLaW, editor. https://www.nav.no/no/nav-og-samfunn/statistikk/sykefravar-statistikk/sykefravar: NAV; 2021.

[9] STAMI. Legemeldt sykefravær [Sickness absence certified by doctor]. In: STAMI, editor. 2019.

[10] Virtanen M, Ervasti J, Head J, Oksanen T, Salo P, Pentti J, et al. Lifestyle factors and risk of sickness absence from work: a multicohort study. Lancet Public Health. 2018;3(11):e545–54. 30409406 10.1016/S2468-2667(18)30201-9 PMC6220357

[11] James SL, Abate D, Abate KH, Abay SM, Abbafati C, Abbasi N, et al. Global, regional, and national incidence, prevalence, and years lived with disability for 354 diseases and injuries for 195 countries and territories, 1990–2017: a systematic analysis for the Global Burden of Disease Study 2017. Lancet. 2018;392(10159):1789–858. 30496104 10.1016/S0140-6736(18)32279-7 PMC6227754

[12] Coenen P, Willenberg L, Parry S, Shi JW, Romero L, Blackwood DM, et al. Associations of occupational standing with musculoskeletal symptoms: a systematic review with meta-analysis. Br J Sports Med. 2018;52(3):176. 27884862 10.1136/bjsports-2016-096795

[13] Lunde L-K, Koch M, Merkus SL, Knardahl S, Wærsted M, Veiersted KB. Associations of objectively measured forward bending at work with low-back pain intensity: a 2-year follow-up of construction and healthcare workers. Occup Environ Med. 2019;76(9):660–7. 31413188 10.1136/oemed-2019-105861 PMC6824615

[14] Swain CTV, Pan F, Owen PJ, Schmidt H, Belavy DL. No consensus on causality of spine postures or physical exposure and low back pain: a systematic review of systematic reviews. J Biomech. 2020;102:109312. 31451200 10.1016/j.jbiomech.2019.08.006

[15] Wærsted M, Koch M, Veiersted KB. Work above shoulder level and shoulder complaints: a systematic review. Int Arch Occup Environ Health. 2020;93(8):925–54. 32572582 10.1007/s00420-020-01551-4 PMC7519900

[16] Gupta N, Rasmussen CL, Forsman M, Søgaard K, Holtermann A. How does accelerometry-measured arm elevation at work influence prospective risk of long-term sickness absence? Scand J Work Environ Health. 2022;2:137–47. 10.5271/sjweh.4000 PMC9045232 34839366

[17] Gupta N, Bjerregaard SS, Yang L, Forsman M, Rasmussen CL, Rasmussen CDN, et al. Does occupational forward bending of the back increase long-term sickness absence risk? A 4-year prospective register-based study using device-measured compositional data analysis. Scand J Work Environ Health. 2022;48(8):651–61. 35894796 10.5271/sjweh.4047 PMC10546616

[18] Török E, Clark AJ, Ersbøll AK, Bjorner JB, Holtermann A, Rugulies R, et al. Physical workload, long-term sickness absence, and the role of social capital. Multi-level analysis of a large occupation cohort. Scand J Work Environ Health. 2020;46(4):373–81. 31840767 10.5271/sjweh.3874 PMC8506317

[19] Sluiter JK, de Croon EM, Meijman TF, Frings-Dresen MHW. Need for recovery from work related fatigue and its role in the development and prediction of subjective health complaints. Occup Environ Med. 2003;60(suppl 1):i62–70. 12782749 10.1136/oem.60.suppl_1.i62 PMC1765724

[20] Bratt C, Gautun H. Should I stay or should I go? Nurses’ wishes to leave nursing homes and home nursing. J Nurs Manag. 2018;26(8):1074–82. 29707851 10.1111/jonm.12639

[21] Gebhard D, Herz M. How to address the health of home care workers: a systematic review of the last two decades. J Appl Gerontol. 2023;42(4):689–703. 36440715 10.1177/07334648221141084 PMC9996797

[22] Tjøsvoll SO, Wiggen Ø, Gonzalez V, Seeberg TM, Elez Redzovic S, Frostad Liaset I, et al. Assessment of physical work demands of home care workers in Norway: an observational study using wearable sensor technology. Ann Work Expo Health. 2022;66(9):1187–98. 35959647 10.1093/annweh/wxac052 PMC9664225

[23] Straker L, Mathiassen SE, Holtermann A. The “Goldilocks Principle”: designing physical activity at work to be “just right” for promoting health. Br J Sports Med. 2018;52(13):818–9. 28663212 10.1136/bjsports-2017-097765 PMC6029635

[24] Holtermann A, Mathiassen SE, Straker L. Promoting health and physical capacity during productive work the Goldilocks Principle. Scand J Work Environ Health. 2019;45(1):90–7. 30021029 10.5271/sjweh.3754

[25] Lohne FK, Fimland MS, Holtermann A, Mathiassen SE, Fischer H, Gellein TM, Redzovic S. Can home care work be organized to promote musculoskeletal health for workers? Study protocol for the Norwegian GoldiCare cluster randomized controlled trial. BMC Health Serv Res. 2022;22(1):1490. 36476502 10.1186/s12913-022-08916-0 PMC9727949

[26] World Health Organization. How to use the ICF: a practical manual for using the international classification of functioning, disability and health (ICF). 2013.

[27] Strand P. Anbefaling fra arbeidsgruppe revidering av IPLOS samlemål [Recommendation from the working group on the revision of IPLOS collective score]. Helsedirektoratet: Helsedirektoratet; 2010.

[28] Lohne FK, Fimland MS, Rasmussen CL, Liaset IF, Fischer H, Redzovic S. Is patients’ activities of daily living self-care score in Norwegian home care a proxy for workers standing at work? BMC Health Serv Res. 2024;24(1):565. 38724977 10.1186/s12913-024-10897-1 PMC11080116

[29] Väisänen V, Ruotsalainen S, Säynäjäkangas P, Mänttäri S, Laitinen J, Sinervo T. Effects of workday characteristics and job demands on recovery from work among Finnish home care nurses: a multi-source cross-sectional study. Int Arch Occup Environ Health. 2024;97(1):65–74. 38032508 10.1007/s00420-023-02026-y PMC10791705

[30] Czuba LR, Sommerich CM, Lavender SA. Ergonomic and safety risk factors in home health care: exploration and assessment of alternative interventions. Work. 2012;42(3):341–53. 22523032 10.3233/WOR-2012-1433

[31] Jacobsen SS, Stevens ML, Karstad K, Rasmussen CDN, Kühnel AB, Holtermann A. A simple resident need-for-physical-assistance scale in Eldercare: validation using 4716 observation sequences of caring activities. Int J Environ Res Public Health. 2022;19(17):10488. 36078204 10.3390/ijerph191710488 PMC9518095

[32] Liaset I, Fimland M, Holtermann A, Mathiassen S, Redzovic S. Can home care work be organized to promote health among the workers while maintaining productivity? An investigation into stakeholders’ perspectives on organizational work redesign concepts based on the Goldilocks Work principles. BMC Health Serv Res. 2023;23(1):667. 37340464 10.1186/s12913-023-09691-2 PMC10283278

[33] Lerche AF, Mathiassen SE, Rasmussen CL, Straker L, Søgaard K, Holtermann A. Development and Implementation of ‘just right’ physical behavior in industrial work based on the goldilocks work principle—a feasibility study. Int J Environ Res Public Health. 2021;18(9):4707. 33925078 10.3390/ijerph18094707 PMC8125316

[34] Ose SO, Lohmann-Lafrenz S, Bernstrøm VH, Berthelsen H, Marchand GH. The Norwegian version of the Copenhagen Psychosocial Questionnaire (COPSOQ III): initial validation study using a national sample of registered nurses. PLoS One. 2023;18(8):e0289739. 37616307 10.1371/journal.pone.0289739 PMC10449149

[35] Burr H, Berthelsen H, Moncada S, Nübling M, Dupret E, Demiral Y, et al. The third version of the Copenhagen psychosocial questionnaire. Saf Health Work. 2019;10(4):482–503. 31890332 10.1016/j.shaw.2019.10.002 PMC6933167

[36] Korshøj M, Skotte JH, Christiansen CS, Mortensen P, Kristiansen J, Hanisch C, et al. Validity of the Acti4 software using ActiGraph GT3X+accelerometer for recording of arm and upper body inclination in simulated work tasks. Ergonomics. 2014;57(2):247–53. 24392673 10.1080/00140139.2013.869358

[37] Skotte J, Korshøj M, Kristiansen J, Hanisch C, Holtermann A. Detection of physical activity types using triaxial accelerometers. J Phys Act Health. 2014;11(1):76–84. 23249722 10.1123/jpah.2011-0347

[38] Stemland I, Ingebrigtsen J, Christiansen CS, Jensen BR, Hanisch C, Skotte J, Holtermann A. Validity of the Acti4 method for detection of physical activity types in free-living settings: comparison with video analysis. Ergonomics. 2015;58(6):953–65. 25588819 10.1080/00140139.2014.998724

[39] Gupta N, Rasmussen CL, Holtermann A, Mathiassen SE. Time-based data in occupational studies: the whys, the hows, and some remaining challenges in Compositional Data Analysis (CoDA). Ann Work Expo Health. 2020;64(8):778–85. 32607544 10.1093/annweh/wxaa056 PMC7544002

[40] Chastin SFM, Palarea-Albaladejo J, Dontje ML, Skelton DA. Combined effects of time spent in physical activity, sedentary behaviors and sleep on obesity and cardio-metabolic health markers: a novel compositional data analysis approach. PLoS One. 2015;10(10):e0139984. 26461112 10.1371/journal.pone.0139984 PMC4604082

[41] Lohne FK, Xu K, Steiro Fimland M, Palarea-Albaladejo J, Redzovic S. Association between musculoskeletal pain and exposures to awkward postures during work: a compositional analysis approach. Ann Work Expo Health. 2024;68(5):522–34. 38603465 10.1093/annweh/wxae027 PMC11285145

[42] Dumuid D, Pedišić Ž, Palarea-Albaladejo J, Martín-Fernández JA, Hron K, Olds T. Compositional data analysis in time-use epidemiology: what, why, how. Int J Environ Res Public Health. 2020;17(7):2220. 32224966 10.3390/ijerph17072220 PMC7177981

[43] Aitchison J, Greenacre M. Biplots of compositional data. J R Stat Soc Ser C. 2002;51:375–92.

[44] Grower J, Lubbe S, Roux Nl. Understanding Biplots. 2011. p. 67–144.

[45] Pawlowsky-Glahn V, Egozcue JJ, Tolosana-Delgado R. Modeling and Analysis of Compositional Data2015. 1–247 p.

[46] Štefelová N, Palarea-Albaladejo J, Hron K, Gába A, Dygrýn J. Compositional PLS biplot based on pivoting balances: an application to explore the association between 24-h movement behaviours and adiposity. Comput Statistics. 2024;39(2):835–63.

[47] Nesrstová V, Jašková P, Pavlů I, Hron K, Palarea-Albaladejo J, Gába A, et al. Simple enough, but not simpler: reconsidering additive logratio coordinates in compositional analysis. SORT (Statistics and Operations Research Transactions). 2023;47:269–94.

[48] R Development Core Team. R: A language and environment for statistical computing. 4.3.1 ed. Vienna, Austria: R Foundation for Statistical Computing; 2023.

[49] Bates D, Mächler M, Bolker B, Walker S. Package Lme4: Linear Mixed-Effects Models Using Eigen and S42014.

[50] Pinheiro J, Bates D, DebRoy SS, Sarkar D. Nlme: Linear and Nonlinear Mixed Effects Models. R package version. 2013;31–110(3):1–113.

[51] Comas-Cufí M. coda.base: A Basic Set of Functions for Compositional Data Analysis. 2023.

[52] Husson F, Josse J, Lê S. FactoMineR: an R package for multivariate analysis. J Stat Softw. 2008;25.

[53] Kassambara A, Mundt F. factoextra: extract and visualize the results of multivariate data analyses. 2020.

[54] Campbell MK, Piaggio G, Elbourne DR, Altman DG. Consort 2010 statement: extension to cluster randomised trials. BMJ: British Medical Journal. 2012;345:e5661. 22951546 10.1136/bmj.e5661

[55] Leff EW, Hagenbach GL, Marn KK. Preventing home health nursing assistant back and shoulder injuries. Jt Comm J Qual Improv. 2000;26(10):587–600. 11042822 10.1016/s1070-3241(00)26050-1

[56] Andersen GR, Bendal S, Westgaard RH. Work demands and health consequences of organizational and technological measures introduced to enhance the quality of home care services – A subgroup analysis. Appl Ergon. 2015;51:172–9. 26154215 10.1016/j.apergo.2015.04.020

[57] Hartvigsen J, Lauritzen S, Lings S, Lauritzen T. Intensive education combined with low tech ergonomic intervention does not prevent low back pain in nurses. Occup Environ Med. 2005;62(1):13. 15613603 10.1136/oem.2003.010843 PMC1740861

[58] Jellema P, Bierma-Zeinstra SM, Van Poppel MN, Bernsen RM, Koes BW. Feasibility of lumbar supports for home care workers with low back pain. Occup Med (Lond). 2002;52(6):317–23. 12361993 10.1093/occmed/52.6.317

[59] Olson R, Wright RR, Elliot DL, Hess JA, Thompson S, Buckmaster A, et al. The COMPASS pilot study: a Total Worker Health™ intervention for home care workers. J Occup Environ Med. 2015;57(4):406–16. 25654631 10.1097/JOM.0000000000000374

[60] Horneij E, Hemborg B, Jensen I, Ekdahl C. No significant differences between intervention programmes on neck, shoulder and low back pain: a prospective randomized study among home-care personnel. J Rehabil Med. 2001;33(4):170–6. 11506215

[61] Roelofs PD, Bierma-Zeinstra SM, van Poppel MN, Jellema P, Willemsen SP, van Tulder MW, et al. Lumbar supports to prevent recurrent low back pain among home care workers: a randomized trial. Ann Intern Med. 2007;147(10):685–92. 18025444 10.7326/0003-4819-147-10-200711200-00004

[62] Proctor E, Silmere H, Raghavan R, Hovmand P, Aarons G, Bunger A, et al. Outcomes for implementation research: conceptual distinctions, measurement challenges, and research agenda. Adm Policy Ment Health. 2011;38(2):65–76. 20957426 10.1007/s10488-010-0319-7 PMC3068522

[63] Farrar JT, Young JP Jr, LaMoreaux L, Werth JL, Poole MR. Clinical importance of changes in chronic pain intensity measured on an 11-point numerical pain rating scale. Pain. 2001;94(2):149–58. 11690728 10.1016/S0304-3959(01)00349-9

