# Supplementary material for: Can home care work be organized to promote musculoskeletal health for workers? Results from the GoldiCare cluster randomized controlled trial
Source: BMC Health Serv Res. 2025 Jan 7;25:41. doi: 10.1186/s12913-024-12133-2 (PMC11708094; doi:10.1186/s12913-024-12133-2)
Supplement: Supplementary file 1 — Additional file 1. GoldiCare – Questionnaire. Translated (Norwegian to English) baseline characteristics questionnaire. [file 12913_2024_12133_MOESM1_ESM.docx]

GoldiCare - Questionnaire

You are invited to participate in the research project 'GoldiCare'. The goal of this project is to try to improve the health of employees in home care services. This questionnaire is an important part of the project, and we kindly ask you to answer as accurately as possible. Please submit the questionnaire in paper form along with the consent form when you attend the data collection (installation of activity monitors) at your unit.

In this questionnaire, you will find job- and health-related questions. If any questions are unclear, you can leave them open and discuss them with the data collection personnel when you attend the data collection session. In some places, we ask about the number of times or length of a period. It may be difficult to remember exactly, so please write the number you think is most accurate.

Every participant is equally important. The more who participate, the more comprehensive and valuable the information we gather will be. In the attached consent form, you will find more detailed information about the project.

Thank you for participating

1. Gender:

_________________

2. Age:

_________________

3. Marital status (Check one):

Single ______
Married ______
Widow/Widower ______
Divorced ______
Separated ______
Registered partner ______
Separated partner ______
Divorced partner ______
Surviving partner ______

4. Which country are you from?

_________________

4a. If you are from a country other than Norway, approximately how long have you lived in Norway?

_______ years

5. What percentage of a full-time position do you hold?

_______% (1 to 100 percent)

6. Do you work shifts, night work, or are you on call? (Check one)

Yes ______
No ______

7. How many years have you worked in home care services?

__________ years

8. Have you had sick leave in the last 12 months? (Check one)

Yes ______
No ______

If yes, how long have you been on sick leave in the last 12 months? (Check one)

Less than 2 weeks ______
More than 2 weeks ______

9. What is your job title? (Check one)

Nurse ______
Nurse assistant ______
Occupational therapist ______
Physiotherapist ______
Social educator ______
Other (please specify) __________________

**Your work**

**How often do you not have time to complete all your work tasks?**
Always Often Sometimes Seldom Never/hardly ever
☐ ☐ ☐ ☐ ☐

**Do you get behind with your work?**Always Often Sometimes Seldom Never/hardly ever
☐ ☐ ☐ ☐ ☐

**Do you have to work very fast?**Always Often Sometimes Seldom Never/hardly ever
☐ ☐ ☐ ☐ ☐

**Do you work at a high pace throughout the day?**
To a very large extent To a large extent Somewhat To a small extent To a very small extent
☐ ☐ ☐ ☐ ☐

**Do you have to deal with other people’s personal problems as part of your work?**
Always Often Sometimes Seldom Never/hardly ever
☐ ☐ ☐ ☐ ☐

**Is your work emotionally demanding?**
To a very large extent To a large extent Somewhat To a small extent To a very small extent
☐ ☐ ☐ ☐ ☐

**Do you have a large degree of influence on the decisions concerning your work?**
Always Often Sometimes Seldom Never/hardly ever
☐ ☐ ☐ ☐ ☐

**To what extent would you say that your immediate superior ...**

**Is good at planning work planning?**
To a very large extent To a large extent Somewhat To a small extent To a very small extent
☐ ☐ ☐ ☐ ☐

**Is good at solving conflicts?**
To a very large extent To a large extent Somewhat To a small extent To a very small extent
☐ ☐ ☐ ☐ ☐

**How often do you get help and support from your immediate superior, if needed?**Always Often Sometimes Seldom Never/hardly ever
☐ ☐ ☐ ☐ ☐

**How often do you get help and support from your colleagues, if needed?**
Always Often Sometimes Seldom Never/hardly ever
☐ ☐ ☐ ☐ ☐

**The next questions are not about your own job but about the workplace as a whole.**

**Do the employees in general trust each other?**
To a very large extent To a large extent Somewhat To a small extent To a very small extent
☐ ☐ ☐ ☐ ☐

**Does the management trust the employees to do their work well?**
To a very large extent To a large extent Somewhat To a small extent To a very small extent
☐ ☐ ☐ ☐ ☐

**Can the employees trust the information that comes from the management?**To a very large extent To a large extent Somewhat To a small extent To a very small extent
☐ ☐ ☐ ☐ ☐

**Are the employees able to express their views and feelings?**
To a very large extent To a large extent Somewhat To a small extent To a very small extent
☐ ☐ ☐ ☐ ☐

**Are conflicts resolved in a fair way?**
To a very large extent To a large extent Somewhat To a small extent To a very small extent
☐ ☐ ☐ ☐ ☐

**Is the work distributed fairly?**
To a very large extent To a large extent Somewhat To a small extent To a very small extent
☐ ☐ ☐ ☐ ☐

WORK ABILITY
How is your current work capacity compared to when it was at its best?

Assume your work capacity at its best is rated as 10. Mark the number that best describes your current work capacity. 0 means you are not able to work at the moment.

Completely unable to work Work capacity at its best

0 1 2 3 4 5 6 7 8 9 10

**HEALTH AND DAILY LIFE**

How is your current health?

Bad Not too good Good Very good
☐ ☐ ☐ ☐

How strong bodily pain have you had the last 4 weeks?

None Very weak Weak Moderate Strong Very strong
☐ ☐ ☐ ☐ ☐ ☐

**Fatigue**

Do you continuously feel fatigued/tired?

Yes No
☐ ☐

If yes: Approximately for how long have you felt fatigued/tired?

Less than 3 months 3-6 months More than 6 months
☐ ☐ ☐
