# Supplementary material for: Can home care work be organized to promote musculoskeletal health for workers? Results from the GoldiCare cluster randomized controlled trial
Source: BMC Health Serv Res. 2025 Jan 7;25:41. doi: 10.1186/s12913-024-12133-2 (PMC11708094; doi:10.1186/s12913-024-12133-2)
Supplement: Supplementary file 2 — Additional file 2. Activity diary. Translated (Norwegian to English) activity diary which was used by participants to note start of periods (work, leisure, sleep, waking up) and their pain in neck/shoulder, lower back and fatigue at the end of the workday. [file 12913_2024_12133_MOESM2_ESM.docx]

**Activity diary for 24-hour measurements**

| Project ID: | Pre **□**  Post **□** |
| --- | --- |

**Please register time (e.g. 13:58) when you woke up, when you arrived for work, when your workday ended, and when you went to bed. If you did not have work for the day, only note time of waking up and going to bed. Also note if any sensors were detached.**

**Contact xxxxxxxxxxxxxxxxx if any questions arise by email: xxxxxxxxxx or phone: xxxxxxxxxx**

| **Date** | **Activity** | **Time (start)** | **Note** (Sensors fell of, sick, etc.) |
| --- | --- | --- | --- |
| **Day 1**  **___ /___** | Sensors attached |  |  |
|  | Finished workday |  |  |
|  | How much pain did you have in the neck/shoulder at the end of this working day? | | 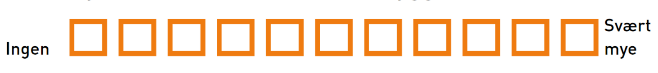 None  Severe |
|  | How much pain did you have in the lower back at the end of this working day? | | 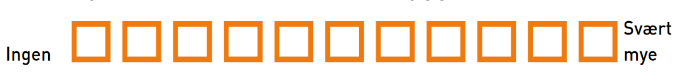 None  Severe |
|  | How tired were you at the end of this working day? | | 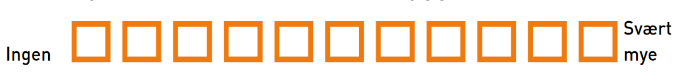 None  Severe |
|  | In bed, ready to sleep |  |  |
| **Day 2**  **____/____** | Woke up |  |  |
|  | Arrived at work |  |  |
|  | Finished workday |  |  |
|  | How much pain did you have in the neck/shoulder at the end of this working day? | | 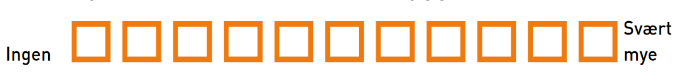 None  Severe |
|  | How much pain did you have in the lower back at the end of this working day? | | 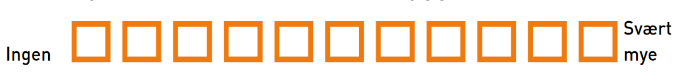 None  Severe 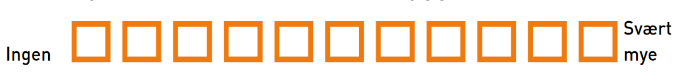 None  Severe |
|  | How tired were you at the end of this working day? | | 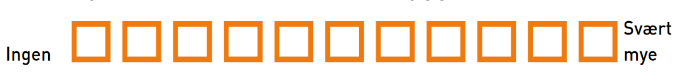 None  Severe |
|  | In bed, ready to sleep |  |  |
| **Day 3**  **____/____** | Woke up |  |  |
|  | Arrived at work |  |  |
|  | Finished workday |  |  |
|  | How much pain did you have in the neck/shoulder at the end of this working day? | | 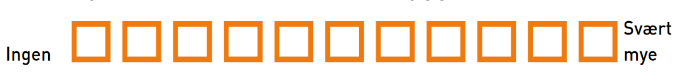 None  Severe |
|  | How much pain did you have in the lower back at the end of this working day? | | 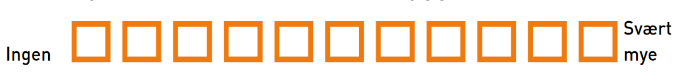 None  Severe |
|  | How tired were you at the end of this working day? | | 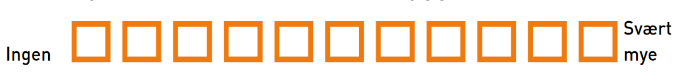 None  Severe |
|  | In bed, ready to sleep |  |  |
| **Day 4**  **____/____** | Woke up |  |  |
|  | Arrived at work |  |  |
|  | Finished workday |  |  |
|  | How much pain did you have in the neck/shoulder at the end of this working day? | | 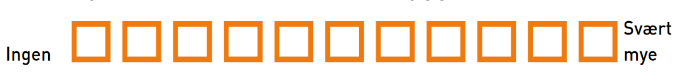 None  Severe |
|  | How much pain did you have in the lower back at the end of this working day? | | 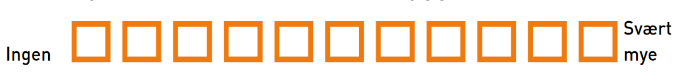 None  Severe |
|  | How tired were you at the end of this working day? | | 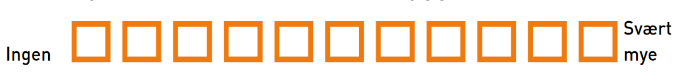 None  Severe |
|  | In bed, ready to sleep |  |  |
| **Day 5**  **____/____** | Woke up |  |  |
|  | Arrived at work |  |  |
|  | Finished workday |  |  |
|  | How much pain did you have in the neck/shoulder at the end of this working day? | | 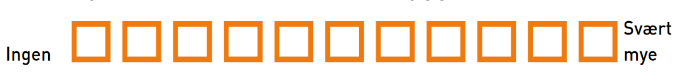 None  Severe |
|  | How much pain did you have in the lower back at the end of this working day? | | 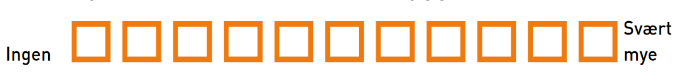 None  Severe |
|  | How tired were you at the end of this working day? | | 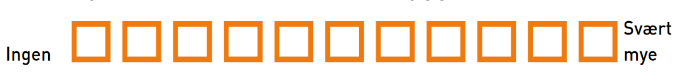 None  Severe |
|  | In bed, ready to sleep |  |  |
| **Day 6**  **____/____** | Woke up |  |  |
|  | Arrived at work |  |  |
|  | Finished workday |  |  |
|  | How much pain did you have in the neck/shoulder at the end of this working day? | | 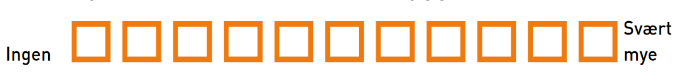 None  Severe |
|  | How much pain did you have in the lower back at the end of this working day? | | 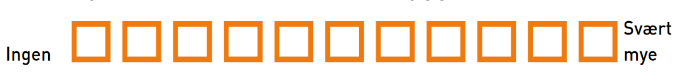 None  Severe |
|  | How tired were you at the end of this working day? | | 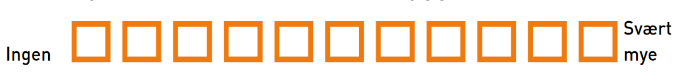 None  Severe |
|  | In bed, ready to sleep |  |  |
| **Day 7**  **____/____** | Woke up |  |  |
|  | Arrived at work |  |  |
|  | Finished workday |  |  |
|  | How much pain did you have in the neck/shoulder at the end of this working day? | | 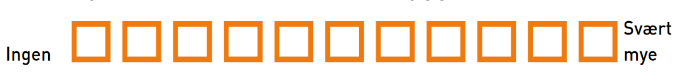 None  Severe |
|  | How much pain did you have in the lower back at the end of this working day? | | 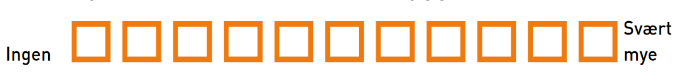 None  Severe |
|  | How tired were you at the end of this working day? | | 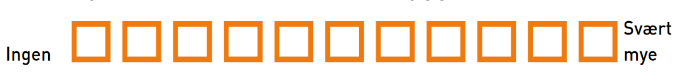 None  Severe |
|  | In bed, ready to sleep |  |  |
