# Supplementary material for: Can home care work be organized to promote musculoskeletal health for workers? Results from the GoldiCare cluster randomized controlled trial
Source: BMC Health Serv Res. 2025 Jan 7;25:41. doi: 10.1186/s12913-024-12133-2 (PMC11708094; doi:10.1186/s12913-024-12133-2)
Supplement: Supplementary file 3 — Additional file 3. Sensitivity analysis results. Results from sensitivity analysis of pain and fatigue, as well as visualization of behaviors and posture compositions from sensitivity analysis. [file 12913_2024_12133_MOESM3_ESM.docx]

**Title**

**Can home care work be organized to promote musculoskeletal health for workers? Results from the GoldiCare cluster randomized controlled trial.**

Author list

Fredrik Klæboe Lohne*, PhD (1,2), Marius Steiro Fimland, PhD (1,2), Javier Palarea-Albaladejo, PhD (4), Svend Erik Mathiassen, PhD (5), Andreas Holtermann, PhD (2), Skender Redzovic, PhD (1)

*: Corresponding author, [Fredrik.k.lohne@ntnu.no](mailto:Fredrik.k.lohne@ntnu.no)

1. Department of Neuromedicine and Movement Science, Faculty of Medicine and Health Sciences, Norwegian University of Science and Technology, Edvard Griegs gate 8, 7030, Trondheim, Norway.
2. National Research Centre for the Working Environment, Lersø Parkallé 105, DK-2100, Copenhagen, Denmark.
3. Unicare Helsefort Rehabilitation Centre, Hysnesveien 11, 7112 Rissa, Norway.
4. Department of Computer Sciences, Applied Mathematics and Statistics, University of Girona, Girona, Spain
5. Department of Occupational Health, Psychology and Sports Sciences, University of Gävle, 80176, Gävle, Sweden

Table S1: *Overall difference in pain and fatigue score (0-10), from baseline to follow-up measurements, along with the difference between intervention group and control group, including only participants from units with >80% adherence to the intervention protocol.*

| Sensitivity analysis | | | | | | | |
| --- | --- | --- | --- | --- | --- | --- | --- |
|  |  | Intervention | | Control | | Intervention effect | |
|  |  | Mean | 95% CI | Mean | 95% CI | Mean | 95% CI |
| NSP | Baseline | 1.80 | 0.93 to 2.68 | 1.64 | 1.00 to 2.28 | 0.12 | -0.43 to 0.66 |
|  | Follow-up | 2.00 | 1.11 to 2.89 | 1.72 | 1.08 to 2.36 |  |  |
| LBP | Baseline | 1.56 | 0.55 to 2.57 | 1.61 | 0.85 to 2.37 | 0.22 | -0.25 to 0.68 |
|  | Follow-up | 1.82 | 0.81 to 2.83 | 1.66 | 0.90 to 2.42 |  |  |
| Fatigue | Baseline | 3.49 | 2.68 to 4.30 | 3.54 | 2.97 to 4.11 | 0.33 | -0.38 to 1.06 |
|  | Follow-up | 3.99 | 3.10 to 4.35 | 3.71 | 3.10 to 4.32 |  |  |


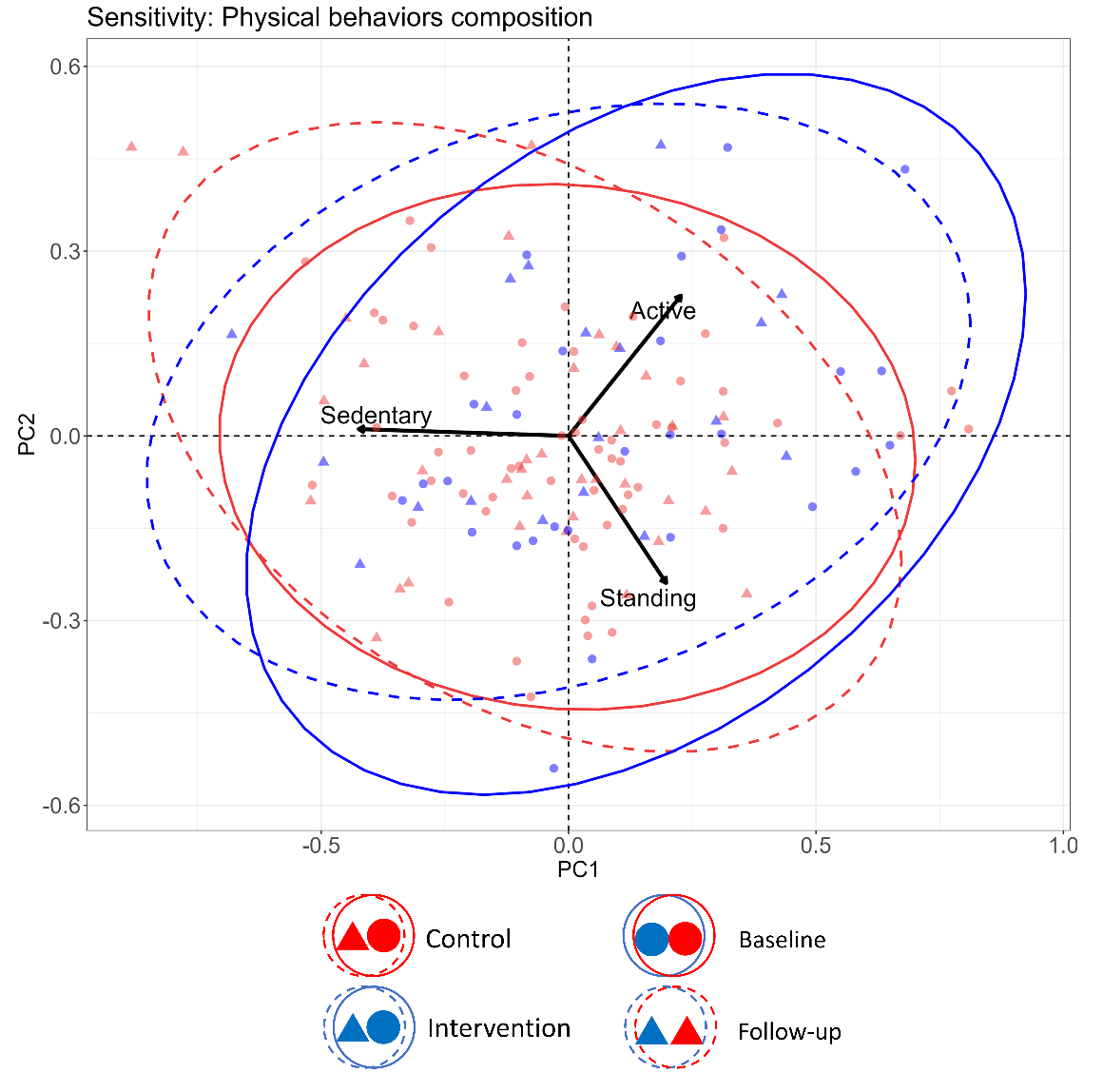


Figure S1: *Compositional biplots illustrating the composition of physical behaviors of the intervention (blue) units with >80% adherence and control group (red) at baseline (fully drawn curves) and after the intervention (dashed curves). The center of the graph (x = 0, y = 0) represents the average composition of the groups. Individual points represent each worker’s weekly mean composition (sedentary, standing, and active). Ellipses illustrate 95% confidence regions for the variance within each group and timepoint (the tighter the ellipse the lower the variability). Arrowheads indicate the most prevalent posture in the respective direction.*


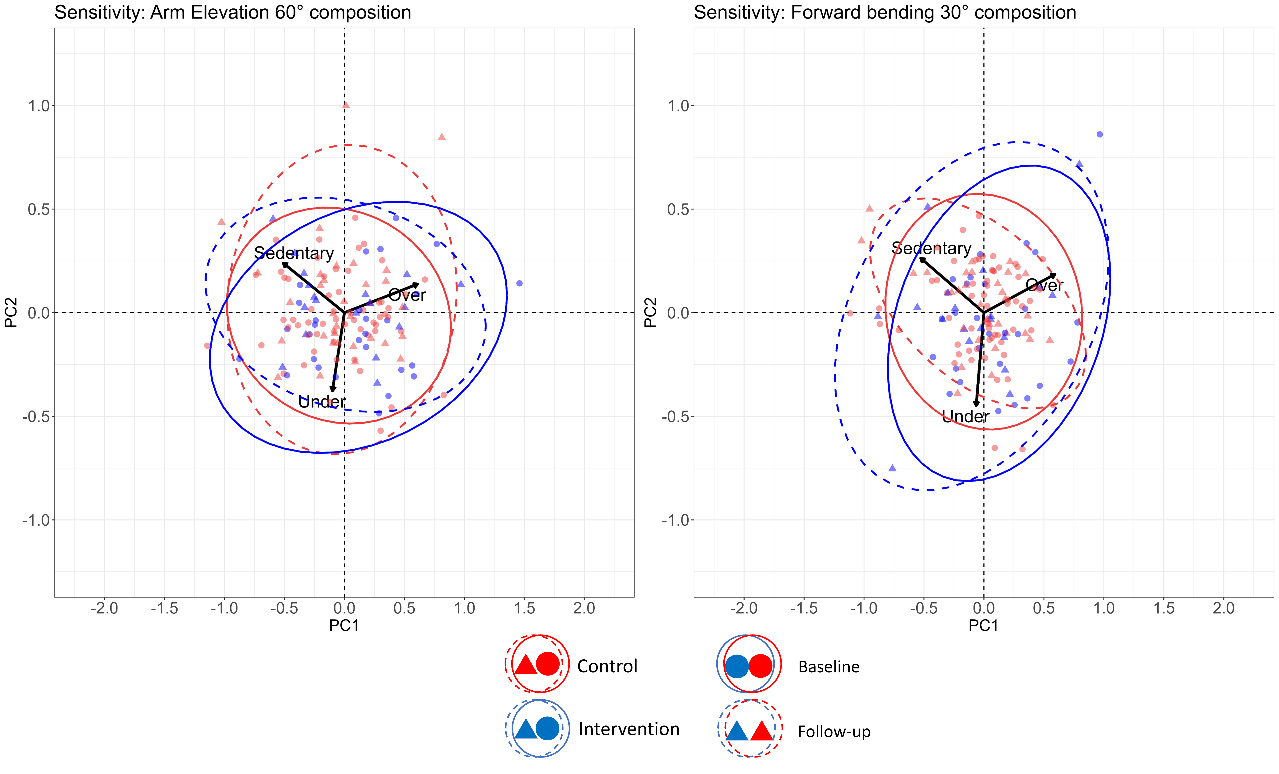


Figure S2: *Compositional biplots illustrating the composition of arm elevation (left panel) and trunk forward bending (right panel) of the intervention (blue) units with >80% adherence and control group (red) at baseline (fully drawn curves) and after the intervention (dashed curves). The graph centers (x = 0, y = 0) represent the average composition of the groups. Individual points represent each worker’s weekly mean composition of arm elevation (sedentary, upright ≤ 60°, upright > 60°) and trunk forward bending (sedentary, upright ≤ 30°, upright > 30°). Ellipses represent 95% confidence regions, illustrating the variability within each group and timepoint (the tighter the ellipse the lower the variability). Arrowheads indicate the most prevalent posture in the respective direction.*
